# Supplementary material for: Profiling of Tumour-Infiltrating Lymphocytes and Tumour-Associated Macrophages in Ovarian Epithelial Cancer—Relation to Tumour Characteristics and Impact on Prognosis
Source: Int J Mol Sci. 2024 Apr 20;25(8):4524. doi: 10.3390/ijms25084524 (PMC11049869; doi:10.3390/ijms25084524)
Supplement: Supplementary file 1 [file ijms-25-04524-s001.zip › ijms-2942721-supplementary.pdf]

Supplementary Table S1. Crude infiltration percentage per HPF according to histological subtype for CD3, CD20, CD68.

		N	Mean	95% Confidence Interval for Mean	
				Lower Bound	Upper Bound
CD3 %	1	15	11.333	3.427	19.239
	2	18	10.028	1.698	18.358
	3	6	11.250	-.687	23.187
	4	12	11.375	4.405	18.345
	5	6	5.000	-2.881	12.881
	6	79	14.918	11.225	18.610
	7	2	6.750	-66.311	79.811
	Total	138	12.873	10.295	15.451
CD20 %	1	15	1.733	.174	3.292
	2	18	.833	.047	1.620
	3	6	2.167	-1.893	6.227
	4	12	.417	-.500	1.334
	5	6	.000	.000	.000
	6	79	1.481	.782	2.180
	7	2	3.000	-22.412	28.412
	Total	138	1.319	.851	1.787
CD68%	1	15	35.667	23.423	47.910
	2	18	31.944	20.498	43.391
	3	6	29.583	2.602	56.565
	4	12	41.250	29.906	52.594
	5	6	21.667	1.299	42.034
	6	79	37.975	33.062	42.888
	7	2	27.500	-67.797	122.797
	Total	138	35.996	32.337	39.656

Supplementary Table S2. ANOVA: One way analysis of variables effect size - infiltration percentage per HPF according to histological subtype for CD3, CD20, CD68.

		Point Estimate	95% Confidence Interval	
			Lower	Upper
CD3 %	Eta-squared	0.031	0.000	0.061
	Omega-squared Fixed-effect	-0.013	-0.045	0.018
CD20 %	Eta-squared	0.037	0.000	0.073
	Omega-squared Fixed-effect	-0.007	-0.045	0.030
CD68%	Eta-squared	0.040	0.000	0.077
	Omega-squared Fixed-effect	-0.004	-0.045	0.035

Eta-squared estimated based on the fixed-effect model.

Negative but less biased estimates are retained, not rounded to zero.
